# Transfer Efficiency and Organization in Turbulent Transport over Alpine Tundra

**DOI:** 10.1007/s10546-024-00879-5

**Published:** 2024-08-28

**Authors:** Laura Mack, Terje Koren Berntsen, Nikki Vercauteren, Norbert Pirk

**Affiliations:** 1 Department of Geosciences, University of Oslo, Blindern, P.O. 1022, 0315 Oslo, Norway; 2 Institute of Geophysics and Meteorology, University of Cologne, Cologne, Germany

**Keywords:** Coherent structures, Eddy-covariance measurements, Organization ratio, Quadrant analysis, Turbulent fluxes

## Abstract

The exchange of momentum, heat and trace gases between atmosphere and surface is mainly controlled by turbulent fluxes. Turbulent mixing is usually parametrized using Monin–Obukhov similarity theory (MOST), which was derived for steady turbulence over homogeneous and flat surfaces, but is nevertheless routinely applied to unsteady turbulence over non-homogeneous surfaces. We study four years of eddy-covariance measurements at a highly heterogeneous alpine valley site in Finse, Norway, to gain insights into the validity of MOST, the turbulent transport mechanisms and the contributing coherent structures. The site exhibits a bimodal topography-following flux footprint, with the two dominant wind sectors characterized by organized and strongly negative momentum flux, but different anisotropy and contributions of submeso-scale motions, leading to a failure of eddy-diffusivity closures and different transfer efficiencies for different scalars. The quadrant analysis of the momentum flux reveals that under stable conditions sweeps transport more momentum than the more frequently occurring ejections, while the opposite is observed under unstable stratification. From quadrant analysis, we derive the ratio of the amount of disorganized to organized structures, that we refer to as organization ratio (OR). We find an invertible relation between transfer efficiency and corresponding organization ratio with an algebraic sigmoid function. The organization ratio further explains the scatter around scaling functions used in MOST and thus indicates that coherent structures modify MOST. Our results highlight the critical role of coherent structures in turbulent transport in heterogeneous tundra environments and may help to find new parametrizations for numerical weather prediction or climate models.

## Introduction

The surface layer is the lowest part of the atmospheric boundary layer in which turbulence is most strongly impacted by the surface and can be regarded as wall-bounded turbulence from a fluid dynamics perspective. Turbulent fluxes in the surface layer are usually described using Monin–Obukhov similarity theory (MOST, Monin and Obukhov (1954)) which states that all dimensionless statistical characteristics of turbulence can be expressed based on the dimensionless stability parameter only. MOST also relates the turbulent fluxes, that are assumed to be constant with height (in the "constant-flux-layer"), to the mean gradients. For the derivation of these properties, MOST assumes homogeneous flat surfaces, quasi-stationarity and that turbulence is generated either mechanically, taking energy from the mean vertical wind gradient (shear-generated) or thermally, taking energy from the mean vertical temperature gradient (buoyancy-generated).

The limitations of MOST result from these strong assumptions: Most surfaces are not flat and homogeneous, leading to a more complex vertical structure of the surface layer (e.g., Babić et al. 2016) accompanied by horizontal gradients due to differential heating, that can induce local circulations (e.g., Lehner and Rotach 2018; Lehner et al. 2021). Such local wind systems and different types of waves, referred to as submeso-scale motions (Acevedo et al. 2014), lead to non-stationarity and non-homogeneity in the flow.

The turbulent fluxes themselves only represent the mean turbulent transport considered in land-atmosphere exchange and thus hide the underlying turbulent structures that actually generate the flux. However, rarely occurring but well-organized structures contribute significantly to turbulent transport (Shaw et al. 1983; Högström and Bergström 1996; Katul et al. 1997; Chowdhuri and Banerjee 2024a) and also the topology of the eddies modifies turbulent transport (Stiperski and Calaf 2018; Chowdhuri et al. 2020; Stiperski and Calaf 2023). The coherent structures transport different quantities with different efficiencies (Li and Bou-Zeid 2011; Guo et al. 2022) and thereby cast doubt on the validity of the "Reynolds analogy" (i.e., momentum and all scalars are transported in the same way) and "Lewis analogy" (i.e., all scalars are transported in the same way Kays et al. 2005). Salesky and Anderson (2020) showed, based on large-eddy simulations, that accounting for velocity fluctuations caused by large coherent structures explains deviations from MOST. However, the effect of coherent structures on MOST is still rarely studied, especially based on eddy-covariance measurements.

We address this gap by studying turbulent fluxes and their transport mechanisms with quadrant analysis, the common method to detect and quantify coherent structures (Thomas and Foken 2007; Wallace 2016), based on eddy-covariance measurements at a heterogeneous tundra site in an alpine valley.

The complex interplay of micro-topography, plants, fungi and soil microbial activity in tundra ecosystems leads to pronounced spatial heterogeneity (Wielgolaski and Godall 1997), which is additionally influenced by a strong seasonal variability in snow cover (Rixen 2022; van der Valk et al. 2022). The investigation of turbulent exchange processes at sites in such heterogeneous terrain is of central importance for model verification and development, but goes along with problems, e.g., a large surface energy balance unclosure (Stoy et al. 2013) and a strong influence of submeso-scale motions on estimates of the CO$_2$ budget (Sievers et al. 2015; Pirk et al. 2017). Therefore, it is crucial to obtain a detailed understanding of the turbulent exchange processes and contributing coherent structures at sites in non-ideal heterogeneous terrain.

We use data from an eddy-covariance site at Finse, Norway, providing four years (from 04/2019 to 03/2023) of almost continuous high-frequency measurements at a single height (Pirk et al. 2023). Our study addresses two aspects: the characteristics of turbulent fluxes in alpine tundra regions specifically, and the influence of coherent structures and their organization on turbulent transport in general, which leads to the two guiding research questions:

- How do turbulent fluxes and transfer efficiencies of momentum, heat, water vapor and CO$_2$ in alpine regions depend on stability?
- How do coherent structures and their organization modify turbulent transport efficiencies?

## Data and Site Description

**Fig. 1:**
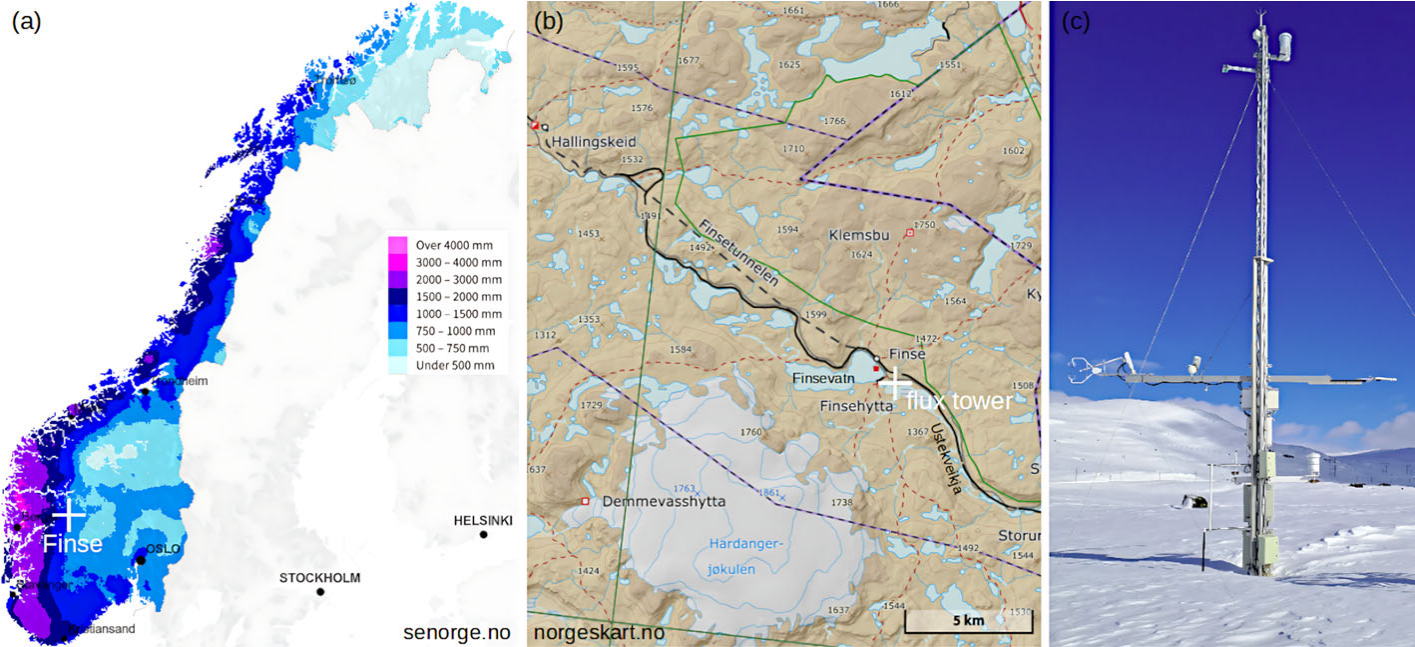
**a** Location of Finse, color-coded mean annual precipitation sum (1991–2020, senorge.no). **b** Map of the valley with flux tower location (norgeskart.no). **c** Flux tower at Finse, photo taken 27.03.2023

Data from a recently established flux tower situated in alpine tundra at Finse, Norway from 04/2019 to 03/2023 are used (Fig. 1a). Finse (60.11$^\circ$N, 7.53$^\circ$E) is located in a valley at the Hardangervidda mountain plateau at about 1220 m a.s.l. (Fig. 1b). The valley extends along a north-west/south-east axis leading to channeling flows with predominant wind directions from west/north-west or east/south-east (Pirk et al. 2023) with on average higher wind speed in winter than summer, the latter a result of the general synoptic situation. The temperature varies from on average -7$^\circ$C in winter to 10$^\circ$C in summer with a total annual precipitation of 980 mm. Snow cover is typically present from October to June reaching its maximum in March with on average 100 cm and maxima far exceeding 200 cm. In winter there is more cloud cover and less cloud-free days than in summer. The surrounding of the Finse site is highly heterogeneous, with the glacier Hardangerjøkulen approximately 6 km south-west, in summer accompanied by a melt water stream, the river Ustekveikja passing the station to the south/south-west and the lake Finsevatn approximately 1 km west of the tower. At more elevated areas and exposed ridges the ecosystem consists of lichen and mountain heath, around the river of moss, floodplains and fens (Bryn and Horvath 2020; Roos et al. 2022). This leads to a diverse footprint (Pirk et al. 2023) and a large surface energy balance unclosure (Pirk et al. 2019; Ramtvedt and Pirk 2022), additionally, affected by a heterogeneous snow melt-out. Due to the insulating effect of the large snow depth, the site is mainly permafrost free (Gisnås et al. 2014).

The flux tower (Fig. 1c) consists of an eddy-covariance system with a CSAT3 three-dimensional sonic anemometer (Campbell Scientific) and a Li-7200 closed-path infrared gas analyzer (Li-Cor) for H$_2$O and CO$_2$ mixing ratios at 4.4 m height. The sampling frequency is 20 Hz. In addition, data from a sonic at 10 m (WindObserver 75, Gill) and a temperature sensor at 10 m (Pt100, Scanmatic and TC Ltd.) are utilized. A detailed site and instrument description can be found in Pirk et al. (2023).

The raw data is processed following the eddy-covariance methodology (e.g., Foken 2017). The data is despiked (based on predefined thresholds, mean-deviation method, and kurtosis and skewness of the averaging interval), a stationarity flag and a tilt correction by double-rotation is applied to the wind measurements. Bandpass-filtering is applied via block averaging in time of a quantity *x*, replacing the ensemble-average assuming ergodicity, defined through:

1 $$\begin{aligned} x = \overline{x} + x' \quad \text {with} \quad \overline{x}:= \frac{1}{t_s} \int _0^{t_s} x(t) \; dt, \end{aligned}$$

with the time average $\overline{x}$ over a time interval $t_s$ and the deviation $x'$ therefrom. Based on multiresolution decomposition (Vickers and Mahrt 2003) of the momentum flux a fixed averaging time of $t_s =$ 30 min was chosen, which fully captures turbulence and is thereby in agreement with assumptions in MOST. This is also the standard approach in similar studies, e.g. Schmutz and Vogt (2019). In contrast, comparing different stabilities while using different averaging intervals (e.g., Stiperski and Calaf 2023) does not allow for a unique attribution of the (later) results to the stability.

## Theoretical Background and Methods

### Turbulent Fluxes and Transfer Efficiency

Turbulence is the main transport mechanism of momentum, heat and trace gases in the atmospheric surface layer. To get a comprehensive picture of turbulent transport at our alpine tundra site, we study turbulent fluxes, the transfer efficiencies and the contributing coherent structures, as detailed in the following.

Using the eddy-covariance method, the turbulent flux and the transfer efficiency of a quantity *x* are given by the covariance and the correlation with the vertical velocity *w*, respectively,

2 $$\begin{aligned} cov(x,w) := \overline{x'w'} \quad \quad \text {and} \quad \quad cor(x,w) := \frac{\overline{x'w'}}{\sigma _x \sigma _w} \in [-1,1]. \end{aligned}$$

The correlation is dimensionless and normalized to the interval $[-1,1]$ due to the normalization with the respective standard deviations $\sigma _x$. Concretely, we will consider the vertical flux of horizontal momentum (*cov*(*u*, *w*) with streamwise velocity *u*), sensible heat (*cov*(*T*, *w*) with temperature *T*), water vapor (*cov*(*q*, *w*) determining the latent heat flux, with specific humidity *q*) and CO$_2$ (*cov*(*c*, *w*) with the CO$_2$ concentration *c*).

### Coherent Structures and Quadrant Analysis

In order to systematically investigate chaotic multi-scale turbulent flows and relate them to dynamical flow properties, coherent structures can be investigated. Coherent structures are structures that retain their characteristics over a statistically significant time period in the order of the integral scale and are very efficient in transport due to their coherence, so that they can lead to large deviations from the mean flow (Jimenez 2018).

**Fig. 2:**
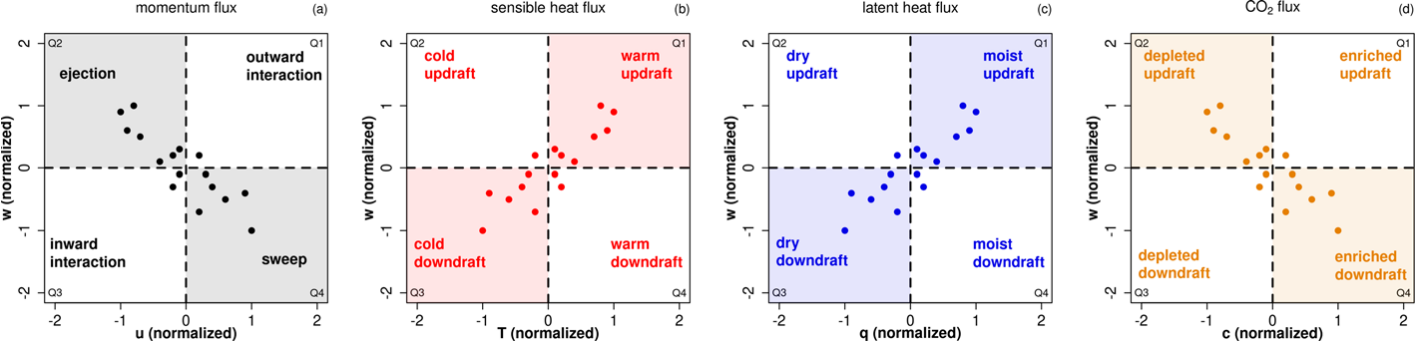
Schematic visualization of the quadrant analysis for **a** momentum flux ($\hat{u},\hat{w}$), **b** sensible heat flux ($\hat{T},\hat{w}$), **c** latent heat flux ($\hat{q},\hat{w}$) and **d** CO$_2$ flux ($\hat{c},\hat{w}$). The shaded quadrants depict "organized" motions, the not shaded ones "disorganized" motions. The dots represent synthetic data points:

Quadrant analysis is a simple conditional sampling method to detect such coherent structures. It can be applied on both eddy-covariance measurements (e.g., Katul et al. 1997) and numerical simulations (as reviewed in detail by Wallace (2016)). The high frequency measurements of a quantity *x* are transformed through

3 $$\begin{aligned} \hat{x}:= \frac{x-\overline{x}}{\sigma _x}, \end{aligned}$$

to a standardized quantity $\hat{x}$ centered around zero. A pairwise scatter plot allows for a classification into four quadrants, as shown in Fig. 2 for momentum flux using $(\hat{u},\hat{w})$, sensible heat flux using $(\hat{T},\hat{w})$, latent heat flux using $(\hat{q},\hat{w})$ and CO$_2$ flux using $(\hat{c},\hat{w})$. By applying the concept of a simple eddy-diffusivity model ("K-theory"), fluxes are assumed to be proportional to the mean vertical gradient. This is used to refer to the two quadrants that contribute to balancing the gradient by having the same sign as the average flux as gradient motions (or organized motions that gain energy from the mean vertical gradient), while the other two quadrants, in which the sign is opposite to the mean flux, are referred to as counter-gradient (or disorganized motions that lose energy to the mean vertical gradient) (Rotach 1993). However, the (infinitesimal) gradient is often uncertain in a discrete measurement tower setup, such that it is unknown if the total flux is gradient or counter-gradient. Therefore, we use a fixed definition of the quadrants as "organized" (shaded, Fig. 2) and "disorganized" (not shaded), which will be related to the correlation later (Sect. 4.3).

The strength (flux contribution) $S_i$ and the time fraction (duration) $D_i$ of a certain quadrant $i \in \{1,2,3,4\}$ are defined by:

4 $$\begin{aligned} S_i:= \frac{\overline{\hat{x}'\hat{w}'}_i}{\overline{\hat{x}\hat{w}}} \quad \quad \text {and} \quad \quad D_i := \frac{1}{t_s} \int _0^{t_s} I_{i}(t) \, dt, \quad \quad I_i := {\left\{ \begin{array}{ll} 1, \text {if } (\hat{x},\hat{w}) \text { in quadrant i} \\ 0, \text {otherwise.} \end{array}\right. } \end{aligned}$$

To measure the ratio of disorganized to organized motions, Shaw et al. (1983) introduced the exuberance *E* as the ratio of the strength of disorganized to organized motions. We define a similar quantity for the time fraction (prevalence), that we call organization ratio *OR*:

5 $$\begin{aligned} OR := \frac{\#\mathrm {(disorganized\,structures)}}{\#\mathrm {(organized\,structures)}}, \, \mathrm {e.g.,\,for\,momentum\,(index\,}uw\mathrm {):}\, OR_{uw} = \frac{D_{uw,1} + D_{uw,3}}{D_{uw,2} + D_{uw,4}}. \end{aligned}$$

*OR* describes the ratio of the amount (#) of disorganized to organized structures and can be defined for any combination of two quantities in the framework of quadrant analysis. If $OR=0$, than no disorganized structures occur, whereas for $OR > 1$ more disorganized than organized structures occur.

### Reynolds Stress Tensor, Turbulent Kinetic Energy and Anisotropy

The Reynolds stress tensor $R:= \overline{u_i ' u_j '}$ describes the shear stresses and its divergence yields to additional unknowns in the Reynolds averaged Navier–Stokes equation. It can be decomposed into an isotropic (direction-independent) and anisotropic part $a_{ij}$ (direction-dependent), whereby the isotropic part is determined by the turbulent kinetic energy $\text {TKE}:= 0.5\,(\overline{u'^2}+\overline{v'^2}+\overline{w'^2})$ (just depending on normal stresses), but only the anisotropic part (containing all shear stresses) contributes to momentum transport (Pope 2000). To generate anisotropy in *R*, at least one of the shear stresses has to be non-zero, i.e., $\overline{u_i'u_j'} \ne 0$ with $i\ne j$, which can only be caused by inhomogeneous flows with a preexisting gradient (Rotta 1951).

From the invariant analysis of *R*, a unique 2-dimensional map to an equilateral triangle called barycentric map (Banerjee et al. 2007) with the two coordinates $(x_B, y_B)$ (with $x_B \in [0,1]$ and a measure for anisotropy $y_B \in [0,\sqrt{3}/2]$) can be derived. The three corners of this triangle represent different limiting states based on the relative magnitudes of the three eigenvalues. Small-scale isotropic eddies (in the 3-component limit) are often assumed to be unaffected by background shear and follow Kolmogorov’s energy cascade (although recent research shows that coherent structures can cause small-scale anisotropy (Chowdhuri and Banerjee 2024b)), whereas larger eddies under strong shear become more anisotropic (Stiperski and Calaf 2018). Without background forcing turbulence returns to isotropy since the pressure-strain term in the equation of motion redistributes energy from velocity components with higher intensity to ones with lower intensity (Rotta 1951). $x_b$ allows to divide anisotropic turbulence further into 2-component (disk-like turbulence) usually associated with strong coherent momentum transport $cov(u,w)<0$ during high shear periods and 1-component (rod-like turbulence) associated with submeso-scale wave-like motions dominating under very stable stratification (Vercauteren et al. 2019).

### Monin–Obukhov Similarity Theory and Flux-Variance Relations

Monin–Obukhov similarity theory (MOST, Monin and Obukhov (1954)) describes turbulence as a balance between buoyancy generation, shear generation and dissipation over flat and homogeneous surfaces and allows to express all dimensionless turbulent quantities based on the dimensionless stability parameter $\zeta$:

6 $$\begin{aligned} \zeta := \frac{z}{L} {\left\{ \begin{array}{ll} < 0 \, \rightarrow \text {unstable} \\ = 0 \, \rightarrow \text {neutral} \\ > 0 \, \rightarrow \text {stable} \end{array}\right. } \quad \text {with} \quad L := -\frac{u_*^3 \, \overline{T}}{\kappa \, g \, \overline{w'T'}} \quad \text {and} \quad u_* := \root 4 \of {\overline{u'w'}^2+\overline{v'w'}^2}, \end{aligned}$$

describing the ratio of measurement height *z* and Obukhov length *L*. *L* is the ratio of shear production (expressed by the friction velocity $u_*$) and buoyancy production (with the von-Kármán-constant $\kappa = 0.4$, gravity acceleration *g* and the heat flux $\overline{w'T'}$). For global scaling the surface fluxes (Monin and Obukhov 1954) and for local scaling the fluxes at the measurement height are considered (Nieuwstadt 1984).

To model turbulent surface fluxes if only the corresponding variance $\sigma _x$ of a quantity *x* is known and the friction velocity derived from it (e.g., Hsieh et al. 1996), Tillman (1972) proposed the flux-variance relation:

7 $$\begin{aligned} \frac{\sigma _x}{x_*} = \varPhi _x(\zeta ) \quad \quad \text {with} \quad \quad x_*:= \frac{\overline{w'x'}}{u_*}, \end{aligned}$$

where $\varPhi _x$ is a stability correction function that has to be derived from observations. Since this function has to apply in the neutral and convective limit it yields the general form $\varPhi _x = a_x (1 + b_x \vert \zeta \vert )^{1/3}$ with the similarity constants $a_x, b_x$ (Panofsky and Dutton 1984). For scalar fluxes an even simpler relation can be used when assuming a complete independence of $u_*$ resulting in $\varPhi _x = a_x \, \vert \zeta \vert ^{-1/3}$ with just one similarity constant $a_x$ (Katul et al. 1994). These relations are used in atmospheric dispersion models (Tampieri 2017) and we will analyze their applicability to our alpine site.

## Results

### Fluxes, Transfer Efficiencies and Quadrant Analysis

**Fig. 3:**
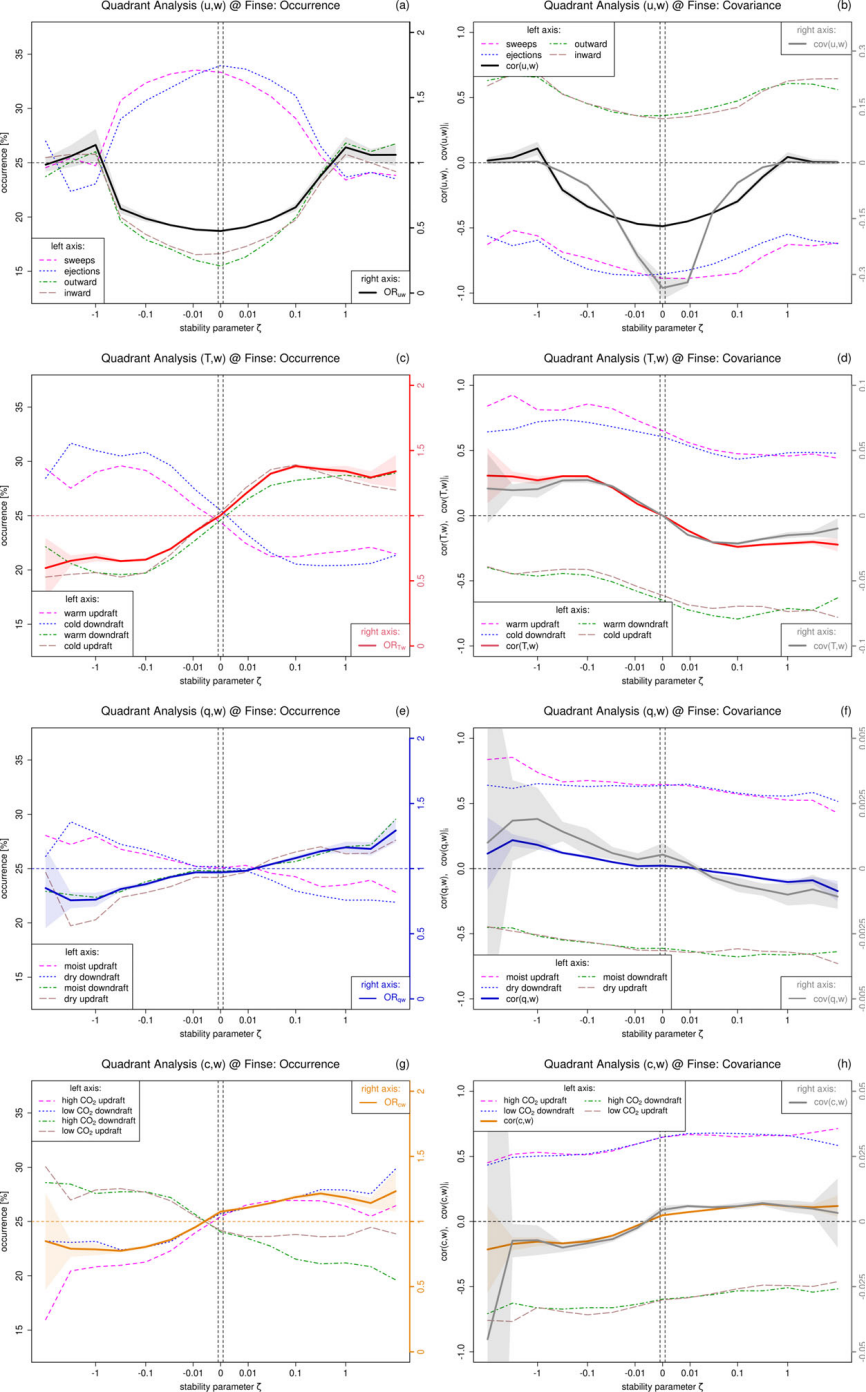
Turbulent fluxes, transfer efficiencies and quadrant analyses of (*u*, *w*) (panels **a**, **b**), (*T*, *w*) (panels **c**, **d**), (*q*, *w*) (panels **e**, **f**) and (*c*, *w*) (panels **g**, **h**). Occurrence frequencies of the four quadrants and organization ratio (**a**, **c**, **e**, **g**). Turbulent fluxes and the contributing quadrants of (**b**) momentum (m$^2$ s$^{-2}$), (**d**) sensible heat (K m s$^{-1}$), (**f**) water vapor (mmol m s$^{-1}$) and (**h**) CO$_2$ ($\mu$mol m s$^{-1}$) and corresponding transfer efficiencies (dimensionless). Thick lines represent the median over all measurements with the uncertainty 5$\, \sigma /\sqrt{\#\text {measurements}}$ (shaded) and the thin lines the median per respective quadrant. Logarithmically equidistant binning of $\zeta$ on $-10^{\{2,1,0,-1,-2\}}$ (unstable) and $10^{\{-2,-1,0,1,2\}}$ (stable)

Figure 3 shows the turbulent fluxes and transfer efficiencies depending on the dimensionless stability parameter $\zeta$ averaged over the four years of measurements at Finse. The contributing coherent structures are investigated by applying quadrant analysis (Sect. 3.2) considering the time fraction of each quadrant and the covariance per quadrant. Note, that we study the nominator $\overline{\hat{x}'\hat{w}'}_i$ of the normalized product $S_i$ directly, since normalizing with close-to-zero fluxes would lead to undefined or highly uncertain values (as discussed in Rotach (1993)).

The momentum flux (Fig. 3b) is strongly negative under near-neutral conditions, indicating shear-generated turbulence, and its absolute value decreases with increasing instability and stability, which follows from the definition of $\zeta$. The transfer efficiency of momentum decreases more slowly with increasing stability and instability, and then suddenly approaches zero for the free-convective limit ($\zeta \rightarrow -1$) and the z-less limit ($\zeta \rightarrow 1$), which can be related to changes in the contributing coherent structures since the organization ratio $OR_{uw}$ (Eq. 5) shows the same stability dependence. This behavior is independent of the applied coordinate rotation and is also observed at the well-studied FLOSS2 (Fluxes Over Snow Surfaces 2) tower (Mahrt and Vickers 2006) for the three lowest measurement levels of 1 m, 2 m and 5 m (but not for higher measurement levels from 10 m upwards). This is a further indicator of the role of disorganized structures that arise from the surface (high values of *OR*) under extreme stabilities (Choi et al. 2004; Li and Bou-Zeid 2011). Under near-neutral conditions, sweeps and ejections are the dominant contributors, each with about 32% occurrence and with a strongly negative flux contribution (Fig. 3a). The organization ratio $OR_{uw}$ reaches the value of $OR_{uw} = 0.5$, i.e., the organized motions occur twice as often as the disorganized. As instability increases, the occurrence time of sweeps and ejections decreases, and inward and outward interactions become more frequent, in agreement with Chowdhuri and Prahba (2019). However, under weakly unstable conditions, sweeps occur more frequently than ejections, but contribute less to the total momentum flux. This suggests a flow with infrequent but very intense and efficient ejections surrounded by many sweeps that carry less momentum. In the convective limit $\zeta \rightarrow -1$, the organization ratio *OR* slightly exceeds one and the total momentum flux approaches zero. Under stable conditions, the occurrence frequency and the contribution of sweeps and ejections decrease again with increasing stability. In contrast to unstable stratification, however, ejections occur more frequently under near-neutral to weakly stable conditions, but contribute less to the total momentum flux. Thus, under stable conditions the momentum flux is carried mainly by infrequent but intense sweeps surrounded by many weak ejections causing intermittent momentum transport associated with recurring bursting referred to as "burst cycle" (Raupach 1981). Our results confirm the findings of Li and Bou-Zeid (2011) under unstable stratification for an alpine site and extend them for stable stratification, revealing an analogy between ejections under unstable stratification and sweeps under stable stratification. The change in the strength and occurrence frequency of coherent structures with stability was further associated – based on both observations (Li and Bou-Zeid 2011) and simulations (Jimenez 2018; Harikrishnan et al. 2021) – to a change in the three-dimensional orientation of the vortex vector from predominantly horizontal under near-neutral conditions (with hairpin vortices that have high horizontal vorticity) to predominantly vertical under unstable conditions (thermal plumes that have high vertical vorticity).

The vertical sensible heat flux (Fig. 3d) is zero under neutral conditions, positive under unstable and negative under stable conditions (as follows from the definition of $\zeta$). Under near-neutral conditions, all four quadrants occur with the same time fraction of 25% (Fig. 3c) and the flux contributions of the quadrants cancel each other out. With increasing instability the organized flows (warm updraft and cold downdraft) occur more frequently. Their time fraction and the total heat flux remain almost constant from $\zeta \approx -0.1$ related to the limited heat transport by large eddies. However, the contribution of warm updrafts to the heat flux continues to increase with increasing instability associated with intense thermal plumes during daytime (Schmutz and Vogt 2019), while their temporal contribution is surpassed by that of cold downdrafts, indicating a flow that contains infrequent and intermittent warm updrafts that transport more heat than the weak but frequent cold downdrafts. This behavior is observed for all measurement heights in the FLOSS2 dataset as well and matches the often observed positive skewness in the probability density distribution of temperature characterizing a long warm tail caused by hot spikes through rising thermals (Tillman 1972). Under stable conditions, almost the opposite behavior is observed: With increasing stability, the time fractions of the disorganized motions (per definition warm downdraft and cold updraft) increase as well as their flux contributions (to the overall negative heat flux). From about $\zeta \approx 0.1$ a plateau is reached again and a further increase in stability suppresses the heat flux which can be used to distinguish weakly from very stable stratification (Mahrt 1998).

The ejection-sweep asymmetry in the momentum flux can also be explained by combining the quadrant analysis for (*u*, *w*) with that for (*w*, *T*) in the framework of octant analysis (e.g., Guo et al. 2022). In unstable stratification sweeps occur in combination with cold downdrafts, while ejections coincide with warm updrafts. Since warm updrafts transport more efficiently than cold downdrafts, although they occur less frequently, it follows that warm ejections are also very efficient in transport, although they occur less frequently than cold sweeps. Under stable conditions, however, sweeps occur with warm downdrafts and ejections with cold updrafts, so that here, due to the more efficient transport of positive temperature perturbations, warm sweeps carry more transport than cold ejections.

The horizontal momentum flux *cov*(*u*, *v*) (not shown) is more than one order of magnitude smaller than *cov*(*u*, *w*), while the horizontal heat flux *cov*(*u*, *T*) is under weakly stable and unstable conditions of the same order of magnitude as the vertical sensible heat flux *cov*(*w*, *T*), which influences the surface energy balance closure (Raabe et al. 2002).

The latent heat flux and its transfer efficiency are positive under unstable conditions (evaporation, sublimation), near zero under neutral, and negative (condensation, deposition) under stable stratification (Fig. 3f). The quadrant analysis for latent heat flux (Fig. 3e,f) shows a similar pattern as the sensible heat flux, in particular moist updrafts contribute most strongly to the latent heat flux under unstable conditions and moist downdrafts under stable conditions (due to the influence of water vapor on buoyancy).

The CO$_2$ flux and its transfer efficiency are negative during unstable stratification (CO$_2$ uptake) and positive during stable stratification (CO$_2$ release) (Fig. 3h). The CO$_2$ quadrant analysis (Fig. 3g,h) shows that under unstable conditions the organized motions are more frequent than the disorganized ones, resulting in $OR_{cw} < 1$, while the opposite is observed under stable stratification.

Overall, the transfer efficiency of sensible heat ($\vert cor(w,T) \vert \le 0.27$) is per stability bin slightly larger than the ones for the other two scalars, water vapor ($\vert cor(w,q) \vert \le 0.19$) and CO$_2$ ($\vert cor(w,c) \vert \le 0.17$). These differences in the transport of scalars, that are contrary to the "Lewis analogy" (e.g., Choi et al. 2004; Kays et al. 2005; Li and Bou-Zeid 2011), can be caused by differences in advection or the surface can feature spatially varying sources and sinks for different scalars, which has been exploited for flux partitioning of latent heat flux and CO$_2$ flux into stomatal and non-stomatal sinks and sources (Scanlon and Sahu 2008; Skaggs et al. 2018). The stability dependence of the covariances and correlations of the scalars is very similar and can clearly be related to the organization ratio. The momentum transfer efficiency differs in shape from the flux, which can be explained by the organization ratio. The strong stability dependence of latent heat and CO$_2$ flux represents a major difference to midlatitude sites (Schmutz and Vogt 2019; Li and Bou-Zeid 2011; Katul et al. 1997) and depicts the influence of seasonal variability of snow-vegetation interaction at our alpine site (Pirk et al. 2023). The stability parameter itself follows a seasonal cycle, with the highest stability being reached in February and March, when the snow depths are at their highest, and the largest instability after snow melt-out in August and September.

All together, in the four years of measurements at Finse, the momentum flux was positive in 18 % of the time. The wind speed difference $\varDelta u / \varDelta z$ (between 10 m and 4.4 m) was negative (i.e., wind speed decreases with height) 22% of the time. The momentum flux was counter-gradient oriented 25% of the entire measurement time. The sensible heat flux was 49% of the time positive (indicating unstable stratification), but the vertical temperature gradient $\varDelta T/\varDelta z$ was negative (i.e., temperature decrease with height) only 20% of the time and 50% of the time the sensible heat flux was oriented counter-gradient. This underlines the limited applicability of simple first-order eddy-diffusivity closures for our alpine site, especially regarding the sensible heat flux. This is in agreement with former studies, which showed that negative dissipations referred to as backscatter—that are generally not captured by eddy-diffusivity models—mainly occur during the onset of warm updrafts in thermal plumes (Porté-Agel et al. 2001). During cloud-free summer days (unstable stratification), the differential heating between glacier and tundra ecosystem is likely to induce a cross-valley circulation (e.g., Mott et al. 2020). During stable stratification, counter-gradient sensible heat flux is often related to a decoupling of the near-surface air (e.g., Foken 2023).

### Footprint-Dependence of Turbulence Characteristics

**Fig. 4:**
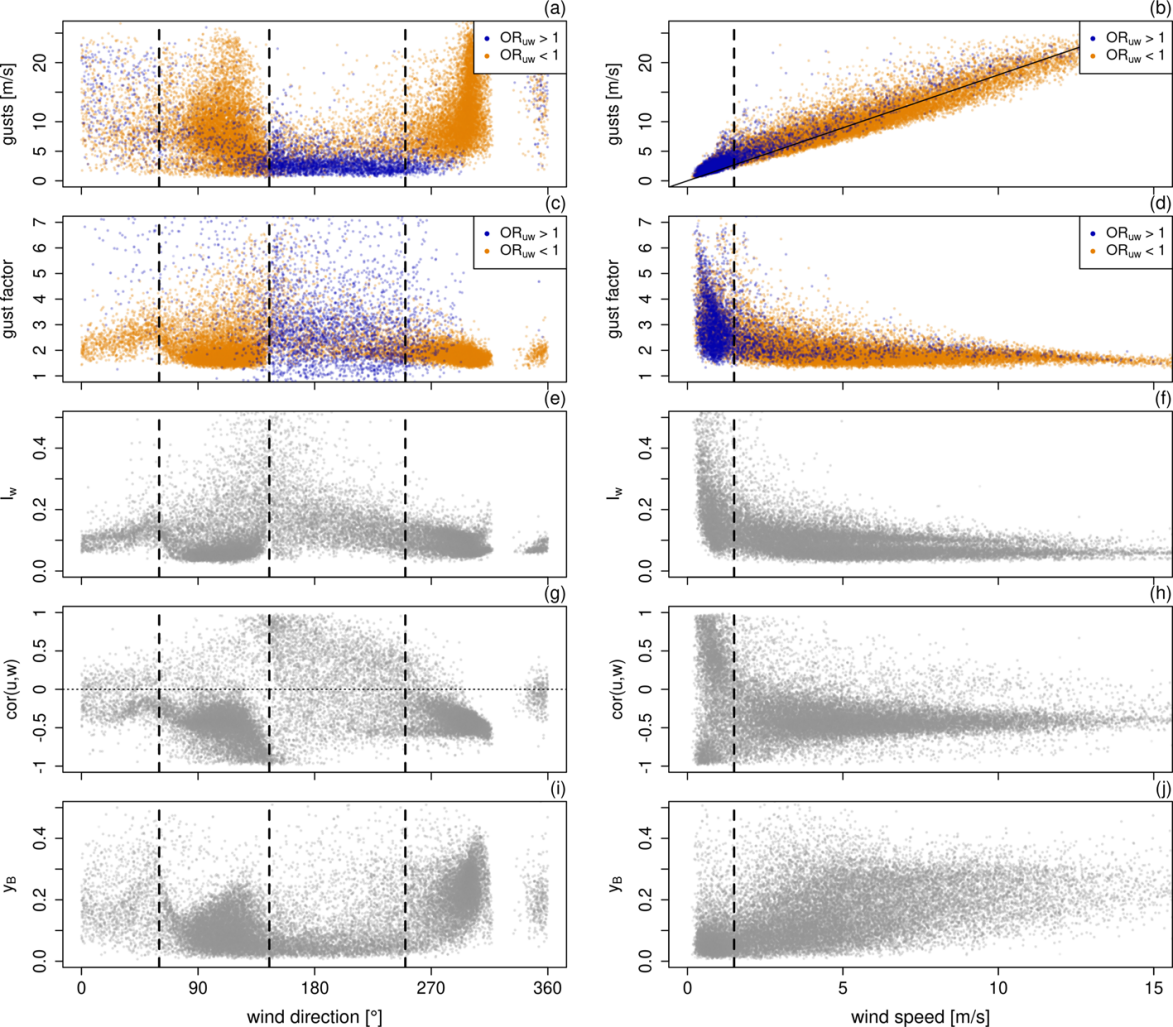
Wind direction (**a, c, e, g, i**) and wind speed (**b, d, f, h, j**) dependence of gusts (**a, b**), gust factor (**c, d**), vertical turbulence intensity $I_w$ (**e, f**), momentum transfer efficiency *cor*(*u*, *w*) (**g, h**) and anisotropy $y_B$ (**i, j**). Color-coding based on momentum organization ratio $OR_{uw}$

Figure 4 shows the wind direction and wind speed dependence of several flow characteristics. The most common wind sectors are east (60–140$^\circ$, 40% of the time) and west (250–310$^\circ$, 35% of the time) corresponding to the valley orientation, followed by the wind sector south (140–250$^\circ$, 18% of the time) oriented towards the glacier and the almost negligible wind sector north (7% of the time). The two main footprints are predominantly caused by the synoptic scale circulation. Easterly winds generally occur in winter when the polar front lies to the south and low pressure systems pass to the south. Oppositely, westerly winds are common when the polar front lies to the north and low pressure systems pass to the north. The southerly wind direction is subject to complex diurnal variations and occurs during winter nights (down-slope katabatic flows from the glacier) and during snow-free summer days (cross-valley down-slope circulation influenced by the glacier).

The valley flow channeling causes higher average wind speeds and stronger gusts (Fig. 4a) along the valley directions (east and west). The maximal gusts of more than 25 m s$^{-1}$ are reached for wind directions from the west related to the usually prevailing synoptic-scale westerly winds. However, the gust factor *G* (Fig. 4c) describing the ratio of gusts to mean wind speed (i.e., $G:= u_{max}/\overline{u}$) is smaller for the two main footprints ($G<2$) and shows large scatter in footprint south reaching values up to $G\approx 7$ despite low gust wind speeds there. The gust wind speed depends linearly on the average wind speed with a slope of 1.792±0.001 (intercept forced to zero, Fig. 4b), whereas the gust factor reaches highest values at low average wind speeds and decreases with increasing wind speed (Fig. 4d). The vertical turbulence intensity $I_w:= \sigma _w/\overline{u}$ (Fig. 4e) shows a similar wind direction and wind speed dependence as the gust factor, which indicates (since both have the same denominator) a close relation between gust wind speed and vertical turbulence fluctuations and thus relates the occurrence of up- and downdrafts to gusts. This is in agreement with Brasseur (2001), who described gusts as a deflection of large-scale winds due to vertical turbulence mixing, and with Suomi et al. (2015), who found that turbulence intensity is the main contributor to the gust factor.

The organization ratio $OR_{uw}$ allows to categorize the wind direction and wind speed dependence into flow regimes. Color-coding (Fig. 4a-d) based on the value of the momentum organization ratio $OR_{uw}$ reveals that the dominant footprints east and west are characterized by organized motions ($OR_{uw}<1$, orange) while disorganized structures ($OR_{uw}>1$, blue) dominate in the footprint south. Up to an average wind speed of 1.6 m s$^{-1}$, the flow is disorganized indicating not fully-developed intermittent turbulence accompanied by high gust factors.

The momentum transfer efficiencies (Fig. 4g) are predominantly negative in the footprints west and east, whereby the most negative values, i.e. most efficient downward transport of horizontal momentum, occur from footprint east. The scatter of the momentum transfer efficiency (and also the scalar fluxes and their corresponding transfer efficiencies, not shown here) is in general higher in the footprint east than west, which may be related to surface characteristics. East of the tower the surface is characterized by a large fraction of late snowbeds and open water, whereas the footprint west is characterized by fens and denser vegetation (Pirk et al. 2023; Bryn and Horvath 2020). In the footprint south the momentum transfer efficiency also reaches positive values (in agreement with the low organization ratio) indicating upward momentum transport, possibly related to negative vertical shear caused by low level jets and secondary circulations initiated through the glacier. Positive momentum fluxes occur only during low wind speeds and with increasing wind speed the momentum transfer efficiency approaches $-$0.5 (Fig. 4h).

Turbulence in Finse is generally highly anisotropic with $y_B <0.4$ during 99 % and $y_B <0.1$ during 36 % of the time. Turbulence in the footprint west is less anisotropic than in the footprint east, whereas the turbulence from footprint south is most anisotropic (Fig. 4i). Anisotropy is highest (low $y_B$) at low wind speeds and tends to decrease with increasing wind speeds (Fig. 4j). At high wind speeds, the turbulence is more isotropic despite consistently negative momentum flux, which is due to a simultaneous increase in TKE with wind speed (e.g., Mahrt et al. 2015; Sun et al. 2012). The Reynolds stress tensor and thus anisotropy are rotation-invariant and therefore unaffected by the applied coordinate rotation in the eddy-covariance post-processing, which is intended to diminish the influence of local topography on the flux calculation. Consequently, anisotropy is affected by the vertical motions due to surface heterogeneity, which at our site are a systematic lifting during easterly winds and sinking during westerly winds. As a result the anisotropy in the footprint east is predominantly caused by ejections, which are more efficient in momentum transport than sweeps there (i.e., $cov(u,w)_2/cov(u,w)_4 > 1$ for footprint east).

**Fig. 5:**
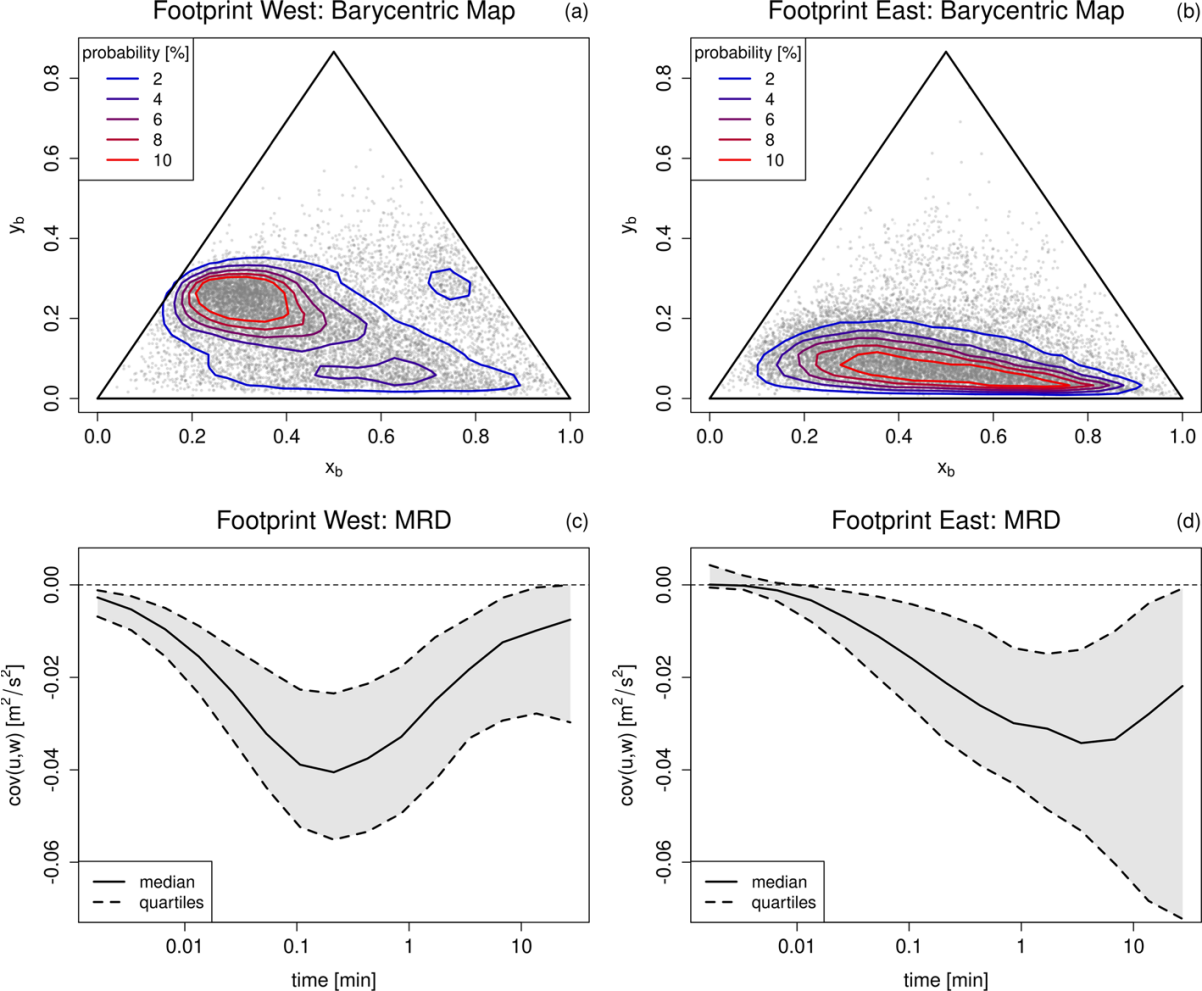
Barycentric maps with $(x_b,y_b)$ for all measurements (dots) and the estimated probabilities (2d kernel density estimation) as contour lines for footprint west (**a**) and east (**b**). MRD cospectrum (up to 30 min) with median (solid) and quartiles (dashed) of momentum flux for footprint west (**c**) and east (**d**)

To investigate whether the anisotropy at our site is also influenced by the actual flow regime (and not just caused by the described vertical motions due to local surface heterogeneity), Fig. 5 shows the barycentric map and the MRD cospectra of the momentum flux. In wind sector west, turbulence is mostly 2-component and occurs on short timescales associated with pure turbulence. However, in the wind sector east the turbulence is more anisotropic and a combination of 1- and 2-component limiting states. The MRD cospectrum is shifted towards larger timescales indicating the importance of submeso-scale motions and shows no clear scale gap between turbulent and submeso-scale motions. The ratio of $cov(u,w)\vert _{\text {30\,min}}/cov(u,w)\vert _{\text {1\,min}}$, that is used to describe the relative importance of submeso-scale to turbulent motions by dividing "full-scale" (30 min) and "small-scale" (1 min) covariance (e.g., Schiavon et al. 2023), is on average three times larger in the footprint east than west. It can be concluded that the anisotropy at our site is not only a result of local topography, but that the anisotropic 1-component limiting state can be related to the occurrence of submeso-scale motions, which have a stronger contribution to the momentum flux in footprint east than west. This is in line with former studies (e.g., Vercauteren et al. 2019; Gucci et al. 2022), which relate the 1-component limiting state to the occurrence of submeso-scale wave-like motions. The momentum flux is 42 % of the time counter-gradient in footprint east, but only 24 % in footprint west, which could be caused by the presence of submeso-scale motions (Acevedo et al. 2014). However, the sensible heat flux is only 37 % of the time counter-gradient in footprint east, but 64 % in footprint west. This indicates a complex interplay of the local topography, different submeso-scale motions and their different ability of transporting momentum and heat.

### Relating Organization Ratio to Correlation

The quadrant analyses (Fig. 3) revealed a strong relation between correlation and corresponding organization ratio. The organization ratio of momentum further allowed to distinguish the predominant wind sectors characterized by organized motions from the less frequent wind sectors characterized by disorganized motions accompanied by high gust factors and strong turbulence intensity (Fig. 4). The *OR*–correlation–relation and the ability of *OR* to relate to failures of classical turbulence closures at our site is exploited in the following.

**Fig. 6:**
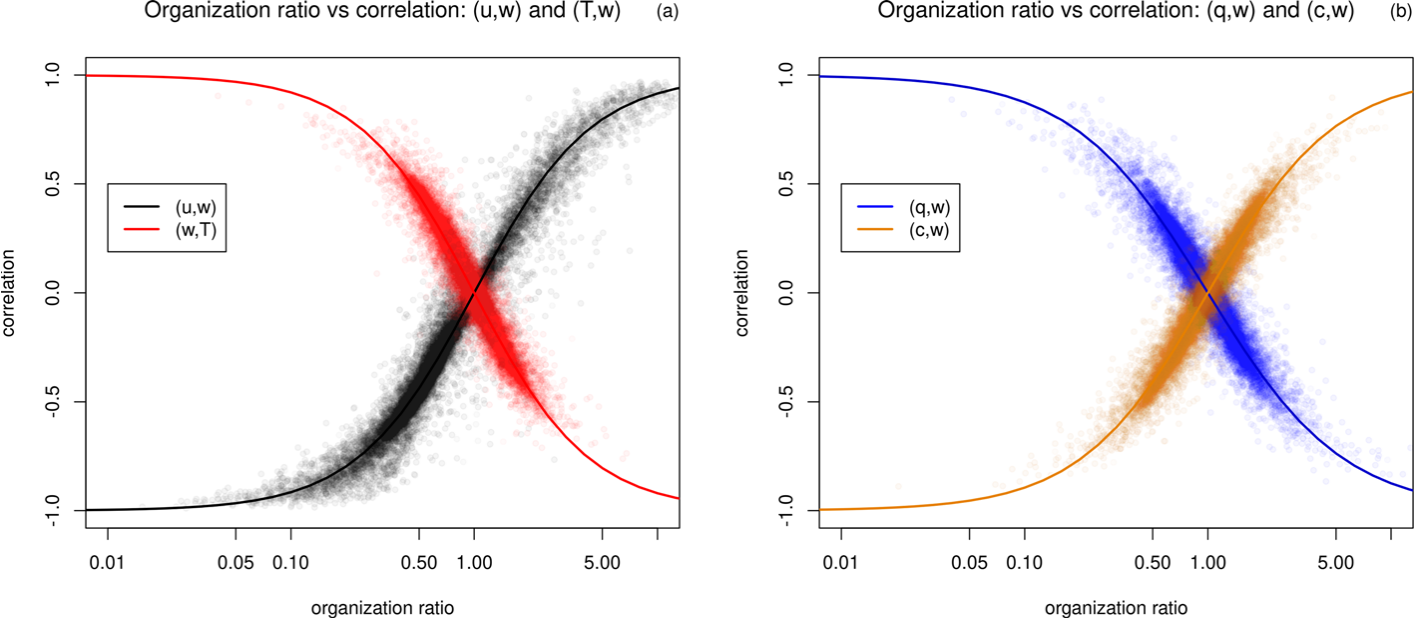
Scatter plot of correlation versus respective organization ratio with the sigmoid fit (Eq. 8) for **a** momentum (black, $s=-1.355 \pm 0.001$) and sensible heat (red, $s=1.382 \pm 0.002$), **b** water vapor (blue, $s=1.175 \pm 0.002$) and CO$_2$ (orange, $s=-1.254 \pm 0.002$)

Figure 6 shows a scatter plot of the correlations against the respective organization ratios for momentum, sensible heat, latent heat and CO$_2$. It can be recognized that the relation between correlation and $\ln (OR)$ follows a logistic sigmoid function, which can be simplified to an algebraic sigmoid function relating the correlation to *OR* through:

8 $$\begin{aligned} cor(x,w) = \frac{2}{1+\big ( OR_{xw} \big )^s}-1, \quad \quad x=u,T,q,c, \end{aligned}$$

with the scaling constant *s* representing the growth rate. The relation follows from the fact that the organization ratio is a binary classification of the areas associated with organized or disorganized motions (Fig. 2). It can be further interpreted as the information loss, if the total correlation $cor(x_1,x_2)$ is described with the correlation for the organized motions $cor(x_1,x_2)\vert _\text {organized}$ only given a certain organization ratio (similar to the concept of mutual information or Kullback–Leibler divergence in information theory). For the momentum transfer efficiency as an example, if $OR = 0$ (i.e., only organized motions), then we can describe the total momentum transfer efficiency with the one for organized motions (sweeps and ejections) without any information loss, however, with increasing *OR* the information loss increases and the total correlation cannot only be described by the one for organized motions.

Furthermore, the organization ratios can be related to the skewness (normalized third-order moment) of the respective turbulent flux in form of $\overline{w'^2x'}$, representing a flux of a flux (Zilitinkevich et al. 1999). $OR_{uw}$ and $OR_{cw}$ correlate positively with the respective third-order moment, indicating a right-tailed distribution for high *OR*, while a negative correlation can be observed for $OR_{wT}$ and $OR_{qw}$ with the respective third-order moment, indicating a left-tailed distribution for high *OR*. This depicts that *OR* (as skewness) measures transport asymmetry (e.g., Wyngaard and Weil 1991) and indicates the role of non-local (and possibly counter-gradient) advective contributions additional to diffusivity (Zilitinkevich et al. 1999).

The concrete scaling constant *s* can be determined from the observations by least-square fits (as listed in the caption of Fig. 6) and is positive for *T*, *q* and negative for *u*, *c*. The absolute value of the growth rate describes the sensitivity of the correlation to changes in the organization ratio. The scatter around the sigmoid fit scales with averaging time. Another property of Eq. 8 is its invertibility, such that each correlation can be uniquely assigned to an organization ratio and in particular this applies to all stabilities simultaneously, i.e., for a given *OR* the correlation does not depend on stability anymore. Thus, the scatter around the average correlation per stability bin can be explained by differences in the organization ratio.

If the division of the quadrants in "organized" and "disorganized" (Fig. 2) would be adapted to the sign of the flux, there would be two separate branches (in Fig. 6) converging from the point (1, 0) with increasing *OR* to -1 and 1 following the same sigmoid function. Thus, this redefinition of the quadrants has the disadvantage that the resulting *OR*–correlation–relation is not unique, but at the same time offers no further information gain, so that a fixed flux-sign-independent quadrant definition is more suitable.

### Relating Scatter in Scaling Functions to Organization Ratio

**Fig. 7:**
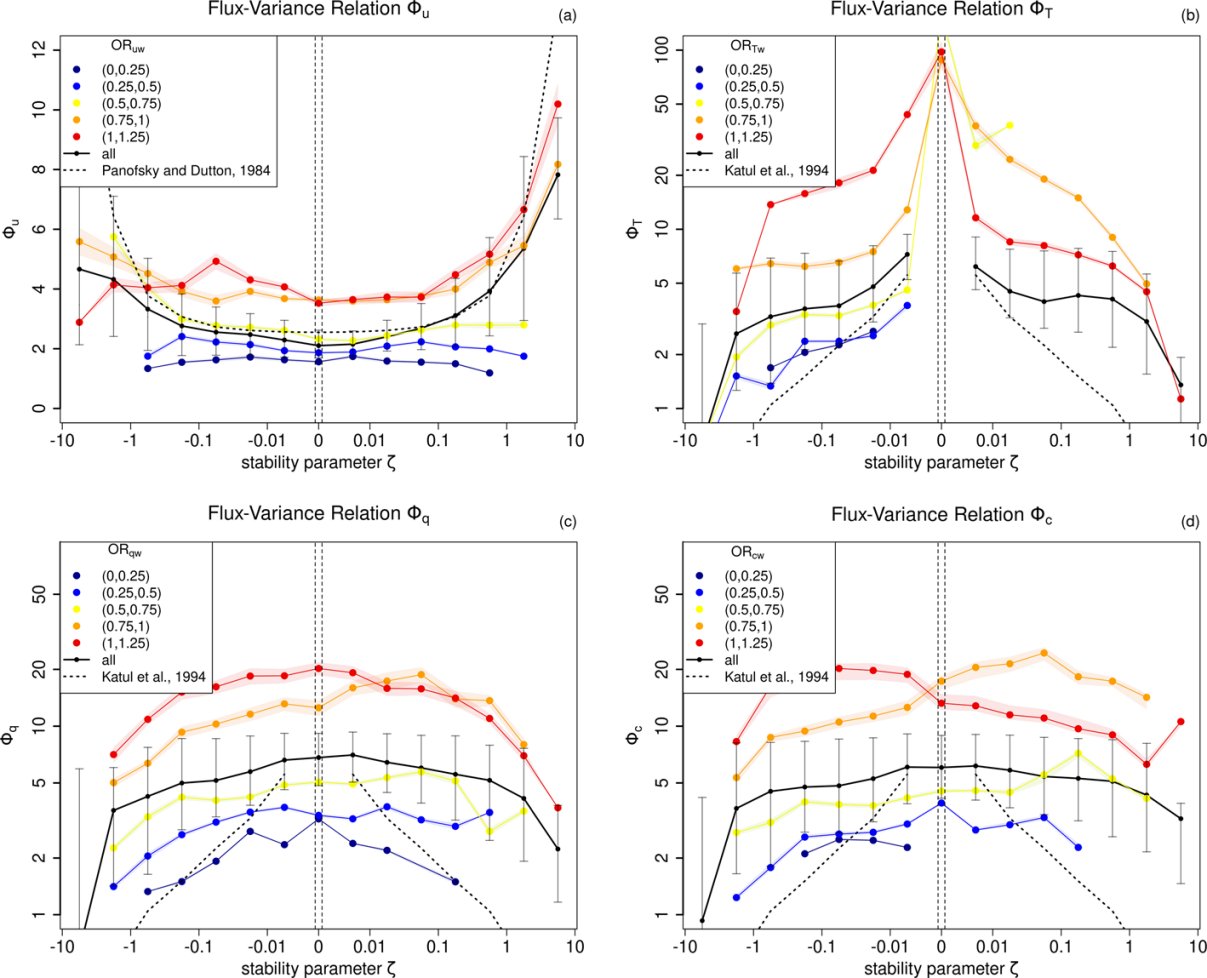
Flux-variance relations $\varPhi _u, \varPhi _T, \varPhi _q, \varPhi _c$ (**a–d**, median in black) with 10 % and 90 % quantiles (error bars) and color-coded the median for different intervals of the organization ratio with estimated uncertainty (5$\, \sigma /\sqrt{\#\text {measurements}}$, color shaded). Dotted lines represent scaling functions from the literature: $\varPhi _u = 2.55 \,(1+3\,|\zeta |^{1/3})$ (Panofsky and Dutton 1984) and $\varPhi _T = \varPhi _q = \varPhi _c = 0.95 \, |\zeta |^{-1/3}$ (Katul et al. 1994). Missing dots indicate that for the corresponding combination of $\zeta$ and *OR* not enough measurements are present

Figure 7 shows the flux-variance relations $\varPhi _u, \varPhi _T, \varPhi _q, \varPhi _c$ averaged (in black) and color-coded for different values of the respective organization ratio *OR*. First of all, by considering the average values (black), it can be seen that $\varPhi _u$ (Fig. 7a) increases more slowly with increasing $|\zeta |$ than in the function proposed by Panofsky and Dutton (1984). $\varPhi _T$ shows a highly nonlinear behavior with a peak in the neutral limit that is caused by the vanishing sensible heat flux and a persisting surface induced non-zero variance. The shape of $\varPhi _T$ and $\varPhi _q$ (Fig. 7b, c) is very different, which raises the need for different scaling functions, which also require more than one parameter (as introduced in Chap. 3.4 following Katul et al. (1994)) to reflect the nonlinear stability dependence (as e.g., in Stiperski et al. (2019) for $\varPhi _T$).

To not only describe the mean but also the scatter around, the colored dots represent the average $\varPhi$ per stability for different intervals of the respective *OR*. It turns out, that *OR* reflects the large scatter around the respective $\varPhi$, with a larger spread for the scalar quantities *T*, *q*, *c*. For all quantities, $\varPhi$ tends to take on higher values for higher values of *OR*. To some extent, the stability dependence of $\varPhi$, i.e. the slope parameter *s*, changes with *OR*, which is particularly noticeable for $\varPhi _u$.

Using the FLOSS2 data, it was verified that *OR* also explains the scatter around the flux-profile relations, which relate the gradient Richardson number to the dimensionless vertical gradients of wind speed and temperature and are usually used to parametrize turbulence under very stable conditions. This can be traced back to a positive correlation between *OR* and the respective mean vertical gradient (i.e., vertical gradient of mean wind speed, temperature, humidity and CO$_2$ concentration).

Stiperski et al. (2019), Stiperski and Calaf (2023) and Mosso et al. (2024) showed that also anisotropy can be used to describe the scatter around the scaling functions with increasing variance for increasing anisotropy, which we could now relate to a lower degree of organization. Therein, the scatter around all scaling functions was explained with the same parameter (anisotropy, that only depends on properties of the Reynolds stress tensor), while we use for every scaling function the corresponding organization ratio, i.e., different predictors for different scaling functions. However, since the relation between correlation and *OR* is unique and invertible, it allows to predict the correlation independently of stability and height, whereas the relation between correlation and anisotropy is not invertible and strongly depends on height (e.g., Stiperski et al. 2019). As already discussed in Chapt. 4.2, anisotropy is rotation-invariant, but variances, transfer efficiencies and fluxes not, such that their relation to anisotropy is rotation-dependent. This could be an advantage of the organization ratio, as the axes of the quadrant analysis itself are rotated by the applied coordinate rotation method. However, both anisotropy and organization ratio are turbulent quantities and therefore cannot directly be used to parametrize subgrid-scale turbulence, but still provide valuable insights into physical reasons for uncertainties in scaling functions.

## Conclusion

We analyze four years of eddy-covariance measurements from an alpine valley site at Finse, Norway. The channeling caused by the valley geometry leads to a bimodal footprint whose main wind sectors (east and west) are characterized by organized and strongly negative momentum flux, while the less frequent wind sectors (south and north) are characterized by disorganized anisotropic flows. Submeso-scale motions occur more frequently in footprint east than west, leading to a significant contribution of the 1-component anisotropic limiting state. The momentum flux is 25 % of the time counter-gradient oriented and the sensible heat flux even 50 %, showing limited applicability of simple first-order eddy-diffusivity closures for our alpine tundra site.

The turbulent fluxes and transfer efficiencies of momentum, heat, water vapor and CO$_2$ show a strong stability dependence, with evaporation and CO$_2$ uptake dominating under unstable conditions, while sublimation/deposition and CO$_2$ emissions dominate under stable conditions. The vertical transfer efficiency of heat exceeds that of latent heat and CO$_2$.

The quadrant analysis of the momentum flux reveals that the organized structures (sweeps and ejections) dominate in terms of both time fraction and strength, under near-neutral conditions, but their occurrence frequency decreases rapidly with increasing instability and stability. Sweeps are more frequent than ejections under unstable conditions, but contribute less to the total momentum flux, while under stable conditions ejections are more frequent than sweeps, but contribute less to the overall momentum flux, characterizing the intermittent nature of turbulent flows under extreme stabilities.

Based on the quadrant analysis, we define the organization ratio (*OR*) as the fraction of the number of disorganized structures to organized structures and show that the relation between organization ratio and transfer efficiency (for all fluxes) follows an algebraic sigmoid function. Since the sigmoid function is monotonic and invertible, the correlation can be uniquely attributed to an organization ratio. Thus, the organization ratio explains the scatter in the flux-variance relations and flux-profile relations used in numerical weather prediction models. Furthermore, *OR* correlates with the respective skewness of the turbulent flux (flux of flux).

Our study contributes to a better understanding of turbulent fluxes at sites in heterogeneous alpine tundra environments characterized by strong seasonal variability in snow cover, a complex footprint and multiscale flows. These factors influence coherent structures and their organization, introducing transport asymmetry (skewness) in turbulent fluxes, which leads to deviations around stability correction functions used in classical turbulence closures. Our results may help to improve turbulence parametrizations, e.g., by representing the occurrence frequency of coherent structures through a stochastic process taking their statistical higher-order properties (skewness and thus organization ratio) into account.

## Data Availability

The code is available on Github (https://github.com/noctiluc3nt/Reddy). The post-processed flux data can be made available upon request. The FLOSS2 raw data is available at https://doi.org/10.5065/D6QC01XR.
